# Mapping longitudinal scientific progress, collaboration and impact of the Alzheimer’s disease neuroimaging initiative

**DOI:** 10.1371/journal.pone.0186095

**Published:** 2017-11-02

**Authors:** Xiaohui Yao, Jingwen Yan, Michael Ginda, Katy Börner, Andrew J. Saykin, Li Shen, for the Alzheimer’s Disease Neuroimaging Initiative

**Affiliations:** 1 Center for Neuroimaging, Indiana University, Indianapolis, Indiana, United States of America; 2 Cyberinfrastructure for Network Science Center, Indiana University, Bloomington, Indiana, United States of America; 3 Indiana University Network Science Institute, Indiana University, Bloomington, Indiana, United States of America; 4 School of Informatics and Computing, Indiana University, Indianapolis, Indiana, United States of America; University of North Carolina at Chapel Hill, UNITED STATES

## Abstract

**Background:**

Alzheimer’s disease neuroimaging initiative (ADNI) is a landmark imaging and omics study in AD. ADNI research literature has increased substantially over the past decade, which poses challenges for effectively communicating information about the results and impact of ADNI-related studies. In this work, we employed advanced information visualization techniques to perform a comprehensive and systematic mapping of the ADNI scientific growth and impact over a period of 12 years.

**Methods:**

Citation information of ADNI-related publications from 01/01/2003 to 05/12/2015 were downloaded from the Scopus database. Five fields, including authors, years, affiliations, sources (journals), and keywords, were extracted and preprocessed. Statistical analyses were performed on basic publication data as well as journal and citations information. Science mapping workflows were conducted using the Science of Science (Sci2) Tool to generate geospatial, topical, and collaboration visualizations at the micro (individual) to macro (global) levels such as geospatial layouts of institutional collaboration networks, keyword co-occurrence networks, and author collaboration networks evolving over time.

**Results:**

During the studied period, 996 ADNI manuscripts were published across 233 journals and conference proceedings. The number of publications grew linearly from 2008 to 2015, so did the number of involved institutions. ADNI publications received much more citations than typical papers from the same set of journals. Collaborations were visualized at multiple levels, including authors, institutions, and research areas. The evolution of key ADNI research topics was also plotted over the studied period.

**Conclusions:**

Both statistical and visualization results demonstrate the increasing attention of ADNI research, strong citation impact of ADNI publications, the expanding collaboration networks among researchers, institutions and ADNI core areas, and the dynamic evolution of ADNI research topics. The visualizations presented here can help improve daily decision making based on a deep understanding of existing patterns and trends using proven and replicable data analysis and visualization methods. They have great potential to provide new insights and actionable knowledge for helping translational research in AD.

## Introduction

Alzheimer’s disease (AD), the most common form of dementia, is a devastating neurodegenerative disorder characterized by gradual loss of brain function, especially impacting memory and other key cognitive capabilities. There is currently no FDA-approved treatment that modifies disease progression [1]. According to the 2015 World Alzheimer report [2], there are over 46 million people worldwide living with dementia at a total cost of US $818 billion in 2015, and the incidence of AD throughout the world is expected to double every 20 years. Given the pressing need to find biomarkers to predict future clinical decline and for use as outcome measures in clinical trials, the Alzheimer’s disease neuroimaging initiative (ADNI) [3] was launched in 2003 as a public-private partnership, led by Principal Investigator Michael W. Weiner, MD and funded by the National Institute on Aging (NIA), the National Institute of Biomedical Imaging and Bioengineering (NIBIB), and through generous contributions from many private partners. The primary goal of ADNI has been to test whether serial magnetic resonance imaging (MRI), positron emission tomography (PET), biological markers, and clinical and neuropsychological assessment can be combined to measure the progression of mild cognitive impairment (MCI) and early AD. ADNI began with an initial 5-year study termed ADNI-1 [4]; and was followed by a 2-year extension termed ADNI-GO, and then by a further 5-year competitive renewal termed ADNI-2. ADNI-GO and ADNI-2 extended the original ADNI-1 study to investigate biomarkers at earlier stages of disease progression. A 5-year competitive renewal termed ADNI-3 began on August 1, 2016, and aims to improve clinical trials and deepen the understanding of the progression and pathophysiology of AD, through investigating various signals including cerebrospinal fluid (CSF) biomarkers and functional imaging techniques like arterial spin labeling (ASL) perfusion MRI, task-free functional MRI (TF-fMRI) and others [5]. For up-to-date information, see www.adni-info.org.

Over the past decade, the ADNI research literature has increased substantially. This poses challenges for effectively communicating information about the results and impact of ADNI-related studies. Several review articles [3, 5–13] summarize the scientific findings of ADNI overall and of specific cores (e.g., [6, 8, 11] for the Genetics Core). While Saykin et al. [11], applied basic information visualization techniques (i.e., topical counts over time and word clouds of genes and journals), no systematic science mapping methods have been used in these studies to analyze the scientific progress and impact. To bridge the gap, in this paper, we employ advanced information visualization techniques [14–22] to perform a comprehensive and systematic mapping of the ADNI scientific growth and impact over a period of 12 years, where the ADNI-1, ADNI-GO and ADNI-2 data were used.

In this paper, using publications that acknowledge ADNI funding, we (1) provide basic statistics that show the growth of ADNI publications and their authors and institutions, (2) evaluate citation impact in relation to expected citation counts, (3) construct network of scientific collaborations geospatially, (4) visualize topic areas with their frequency, co-occurrence, and evolution over time, and (5) illustrate evolving of collaboration networks of institutions over time.

Subsequently, we review science of science studies in general and then discuss related work on mapping AD and ADNI data specifically. Next, we describe our materials and methods. After that, we present our results. Finally, we conclude the paper with a discussion and outlook.

## Related work

### Science of science studies

Scientific studies of the dynamics of science, technology, and innovation (STI) aim to improve our understanding of the structures and processes that facilitate the development of usable knowledge; develop theories of creative processes and their transformation into social and economic outcomes; evaluate returns from investments; and examine the impact of policy decisions on the contexts, structures and processes of STI. Relevant studies are performed by scholars in scientometrics, bibliometrics, information science, physics, economics, social science, sociology of science, history of science, and many other fields that have vastly different research cultures, approaches, and tools [15, 16].

More and more science leaders and policy makers use data mining and visualization techniques to gain insights into the structure and evolution of STI to inform their decision making. They are compiling, cleaning and interlinking large-scale publication, patent, and funding datasets and then apply temporal, geospatial, topical, and network analyses and visualizations to understand STI dynamics at the individual (micro) to global (macro) levels. The *Places & Spaces: Mapping Science* exhibits features more than 100 STI visualizations [23]. The Science of Science (Sci2) Tool [21] used in this article was specifically designed for the study of science by scientific means and is widely used in research, teaching, and practice.

### ADNI studies

Data generated by the ADNI project (e.g., MRI, PET, fluid biomarker, genetics, clinical and cognitive data) has greatly facilitated the scientific progress of AD research. Given the availability of a wide variety of ADNI data modalities, many imaging, biomarker and genetics data processing pipelines and analytical approaches have been developed and novel multi-modal disease biomarkers have been identified. Results are written up in more than 996 publications, and meta-studies of the publications have been conducted. For example, in an ADNI special issue of *Alzheimer’s and Dementia*, Weiner et al. [3] summarized the scientific findings and achievements of all the publications that acknowledge the usage of ADNI data over the course of the ADNI project based on their topical focus, e.g., development and assessment of treatments, data processing methods, or data analysis. The same special issue also features reviews for all the ADNI cores, e.g., the MRI core [9], informatics core [12], biomarker core [10]. Taking the ADNI Genetics Core for example, Saykin et al. [11] compiled an extensive dataset of 300 publications published between 2008 and 2014 that used ADNI genetic data to showcase scientific progress of this particular core. Shen et al. [8] reviewed 106 publications between 2009 and 2012 to summarize analytical strategies used in and genetic findings identified through case control studies and association analyses of multi-modal quantitative phenotypes. Recent work by Toga et al. [12] uses web access activities of the online ADNI database to examine the impact of ADNI data on multi-institutional collaborations using line plots and geospatial maps to demonstrate the increasing number and global distribution of data users.

Other work has aimed to show the full scope of AD research. The International Alzheimer’s Disease Research Portfolio (IADRP) [24] is an online database of funded AD projects. Users can perform searches using terms from the Common Alzheimer Disease Research Ontology (CADRO) and/or other information. IADRP provides basic interactive visualizations, e.g., showing numbers of the projects categorized using the CADRO terms. Hughes et al. [25] employed social network analysis and mapped the growth and impact of NIA funded Alzheimer Disease Centers (ADCs). A study analogous to what has been done for ADCs [25] would be particularly valuable for understanding the impact of the ADNI project.

## Materials and methods

This section details data collection and preparation together with the tools, algorithms, and workflows applied.

### Data collection and preparation

A Scopus [26] publication search was performed on 05/12/2015 to identify ADNI-related papers published between 01/01/2003 to 05/12/2015. Publications were required to satisfy both of the following criteria: (a) “ADNI” or “Alzheimer’s disease neuroimaging initiative” were mentioned in title, abstract, keywords, or authors fields; (b) “AG24904” or “AG36535”-the two main ADNI grant award numbers-were listed in the funding number field. Post hoc inclusion criteria are as follows: (1) publications were limited to the following types: articles, reviews, conference papers, or articles in press; and (2) all were written in English language. The final set of publications contained 722 articles, 52 reviews, 196 conference papers, and 26 articles in press.

Subsequently, five fields were extracted for each publication: authors, sources (i.e., journals and conference proceedings), affiliations, keywords, and year. While “year” is an integer value, all the other fields contain text that requires preprocessing to generate standardized terms needed for subsequent analyses and visualizations. The following standardization processes were applied to text.

#### Authors

Author names were disambiguated using a combination of key collision and nearest neighbor clustering algorithms provided by Open Refine [27, 28]. An initial list of similar author names was identified and updated, e.g., “Kunkle B.” vs “Kunkle B.W.” and “Lee J.H.” vs “Lee J.-H.” Next, for each set of similar names, full names were retrieved manually from the original papers. In cases where full names were identical, the names were merged. After that, the cleaned author data were loaded into the Science of Science (Sci2) Tool [21] and the author co-occurrence network was extracted. Using the resulting network output file, a final disambiguation using string similarities among author names were calculated. The resulting outputs were manually reviewed before names were merged, and the final list of unique names was used to derive the final co-author network.

#### Papers with many authors

For papers with 50 or more authors, author lists were truncated by including only the first 25 and last 25 authors with the corresponding affiliations, to avoid extremely dense visualizations on collaboration networks. S1 Fig shows the number of publications against the forward and backward author positions.

#### Sources (journals)

Journal names were standardized using a process analogous to the one described for authors. Potential duplicates (e.g., *Journal of Alzheimer’s Disease* and *J. Alzheimer’s Dis.*) were identified using Open Refine clustering algorithms. Identified duplicates were merged if ISSN was available and matched, or using manual lookup of the title in the SCImago Journal Rank (SJR) data set. Any abbreviated journal names were updated to full names using the Scopus Journal Metrics (SJM) data set. After processing, one journal was identified to have changed titles (*Archives of Neurology* to *JAMA Neurology*) and was given a common set of identifiers for analysis.

#### Affiliations

Each publication records the affiliations of each author; and an author may have multiple affiliations. Multiple instances of a given affiliation string were reduced to one instance per publication to calculate the number of publication collaborations between two affiliations in the network. Open Refine was used for affiliation clean up and name disambiguation. Names were cleaned to maintain only broad institution name (e.g., university name, company name) and location (i.e., city, state, and country) preserved; mailing codes were removed (e.g. *“School of Medicine, Indiana University, Indianapolis, IN, United States, 46202”* became *“Indiana University, Indianapolis, IN, United States”*).

After processing, further disambiguation was required to find duplicate affiliations based on acronyms, the language used to report names and locations, and the use of neighborhood and regional names. First, a co-occurrence network was extracted from the affiliations field in Sci2, which creates a merge list of node labels from the affiliation field. The merged table provides a method to update a network with a unique list of affiliations names from the network in Sci2; each affiliation name is assigned a unique identifier in the “uniqueIndex” field and a token that indicates whether a node is merged during an update in the “combinedValues” field.

To complete affiliation name disambiguation, the merged list was saved and the locations were geocoded using Open Refine and the Google Maps JavaScript Geocoding API [29]. The process successfully geocoded 815 of the 902 affiliations, and the resulting latitude and longitude values were parsed from the API results as a concatenated field of geo-coordinate pairs. The 87 non-geocoded affiliations were then manually searched on Google Maps to identify the remaining latitude and longitude pair values. Nine affiliations did not have sufficient data for geocoding and were excluded from the analysis. Affiliation latitude-longitude pairs with multiple affiliations associated with them were manually reviewed and updated to identify unique affiliations to a location and a representative institution name.

The preliminary network was updated in Sci2 using updated merge table. After updating the network affiliation nodes with geo-coordinates, a geospatial network layout is generated by assigning nodes an XY coordinate based on an affiliations’ latitude and longitude. The XY coordinates correspond to latitude and longitude for a common base map of the world.

### Data analysis and visualization

We first performed analysis on basic data statistics, and then examined journal statistics on expected and actual citations. After that, the Science of Science (Sci2) Tool [21] was used to perform the geospatial, topical, temporal, and collaboration network analysis and visualization workflows at the micro (individual) to macro (global) levels, including affiliation collaborations with geospatial layouts, keyword co-occurrence networks, topic temporal evolution, and author collaborations over time.

#### Basic data statistics

Basic statistics of publications were investigated including the number of papers published over years and the number of institutions involved over years, to demonstrate the growth and impact of ADNI project. We also explored the number of authors and institutions for each paper, to compute and visualize the collaboration statistics.

#### Journal and citation statistics

The Scopus Journal Metrics (SJM) was used to understand and compare citation impact for publications across journal sources [30]. SJM citation metrics used here include: (1) Source Normalized Impact per Paper (SNIP) which measures contextual citation impact by weighting citations based on the total number of citations in a subject field [31]; (2) Impact per Publication (IPP) which measures the ratio of citations per article published in a journal [32]; and (3) SCImago Journal Rank (SJR), a prestige metric where citations’ subject field, quality and reputation of the journal have a direct effect on a journal’s rank [33]. The metrics are produced annually (1999-present) for journals and conference proceedings indexed by Scopus based on a journal sources’ citation patterns over the previous three-year period. That is, the SJM data covers all years of the ADNI data set between 2003 and 2014.

A database was setup to join a table of ADNI publications data and the SJM data tables via the full source name for each publication. A set of queries were run to identify each publication’s IPP, SNIP, and SJR for its publication year. Publications from 2015 were given the 2014 rankings for this analysis. The results were then analyzed and visualized.

#### Networks with geospatial layout

To create a network with a geospatial layout, publication data was loaded into Sci2, and a co-occurrence network was extracted from a field that might have an associated geo-location, e.g., institution address. The process used to geo-locate the ADNI affiliations is described above, which produces a network where all institution nodes have geo-coordinates and hence can be geo-located on a map. Next, the geo-coded network was loaded into Sci2 and the algorithm *Geospatial Network Layout with Base Map* was applied, which produces a base map in post-script format and network with XY coordinates that position nodes to overlay onto the base map after visualization.

The network with geospatial layout was analyzed and visualized using Gephi [34]. The final visualization was produced to show only affiliation nodes associated with 4 or more publications and edges with 4 or more works between two nodes. Both nodes and edges color and size were scaled proportionally using a Bezier curve. The resulting network was exported and Photoshop was used to combine the base map and network layout for visualization.

#### Keyword co-occurrence network

Word co-occurrence network extraction was applied to construct the keyword co-occurrence network in which nodes represent keywords and weighted edges denote the number of times they jointly appeared in a publication. *Network Analysis Toolkit (NAT)* embedded in Sci2 was employed to statistically analyze the network. *DrL algorithm*-one of the few force-directed layout algorithms-was selected to layout the network based on the popular VxOrd routine [35].

The co-occurrence network layout was analyzed and visualized using Guess [36]. Filters were applied to label and show only keyword nodes with degree ≥ 10. Labeled keywords were categorized into four groups, including phenotype, genotype, analysis, and others; and were colored by the corresponding groups.

#### Topic evolution

Twenty-three keywords were provided by a domain expert in AD research to illustrate the temporal evolution of major AD research topics. For each keyword, a normalized frequency was calculated from dividing its actual frequency in a certain year by the total number of publications in the same year. The trajectory of the normalized frequency over years was plotted for each keyword. Hierarchical clustering was performed to identify keywords with similar temporal profiles.

#### Co-publication network over time

To explore the growth of institution collaboration over time, publications were partitioned into time periods. For each time period, co-affiliation network was constructed using co-occurrence network extraction algorithm based on the institutions’ publication collaborations. In the co-publication network, nodes represent institutions, and each edge is weighted by the number of co-occurrences of two corresponding institutions in these publications.

The co-publication network layout was analyzed and visualized using Gephi, where the *Fruchterman Reingold algorithm* [37] was employed for the network. Nodes were colored and scaled proportionally to the natural logarithm of their degrees. Similarly, edges were scaled proportionally to the frequencies of co-publications between their linked nodes based on Bezier curve. For each co-publication network, two statistical measures were computed: 1) the weight of co-publications, which is the total number of co-occurred institution pairs; 2) the degree of co-publications, which is the number of collaborations for each institution. While analyzing these statistics, two situations were considered separately if there were multiple co-publications (i.e., weight of edges > 1) between two institutions: (1) count edges only once (denote as unique edge), or (2) count the actual number of collaborations allowing duplicates (denote as weighted edge).

## Results

### Basic data statistics

**Fig 1:**
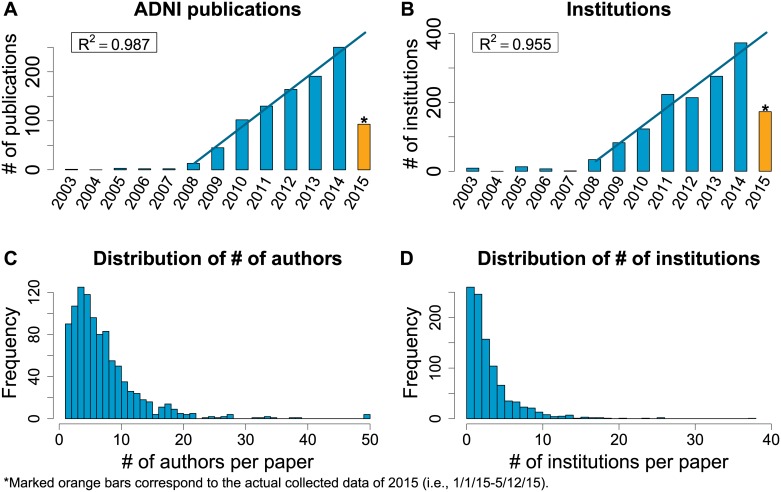
Statistics for ADNI publications between 01/01/2003 to 05/12/2015. (A) Growth of ADNI publications on the year-by-year basis; line indicates a linear regression prediction for the 2015 number using data from 2008 to 2014. (B) Growth of institutions involved in ADNI publications; line indicates a linear regression prediction for the 2015 number using data from 2008 to 2014. (C) Distribution of number of authors per paper. (D) Distribution of number of institutions per paper.

A total number of 996 ADNI papers were published between 01/01/2003 and 05/12/2015, based on a Scopus search performed on 05/12/2015 and a subsequent manual filtering procedure described in the Methods section (see S1 Dataset for processed data). Three papers contained more than 50 authors. To facilitate the following analysis, the author lists of these three papers were truncated to keep only the first 25 and last 25 authors with their corresponding affiliations (see also S1 Fig for a plot of the number of publications against the forward and backward author positions). The basic statistics of all these 996 publications are shown in Fig 1. Starting in 2008, the number of ADNI publications increased linearly with a doubling time of 1.407 year (Fig 1(A)), so did the number of institutions involved in the ADNI publications with a doubling time of 1.736 year (Fig 1(B)). R-squares for both are larger than 0.95. Note that data for 2015 is incomplete. The increase is likely due to the growing attention on the AD research and the fast expanding collaboration networks.

Fig 1(C) and 1(D) show the distributions of the numbers of co-authors and co-institutions across all 996 publications; only truncated author lists are considered for the three papers with more than 50 authors. As we can see, the majority of papers have more than one author, and on average there are 7.8 authors per paper. While 14.86% of papers were written by authors from one institution, all the others reported results by authors from multiple institutions.

### Journal statistics analysis of expected and received citations

Using the results of the journal metrics data preparation methods, the data set was analyzed using the R statistical software [38]. The analysis evaluated the citation impact of ADNI publication to identify significant publication venues that contribute to the citation impact across journal metrics and ranks.

The ADNI publications cut across 233 journals and conference proceedings; see S2 Fig for a few top journals/proceedings. These publications gathered a total of 18,522 citations. ADNI research earned a median of 3 and a mean of 18.6 citations per publication.

Only 859 publications sources had both IPP and SJR journal metrics, which cover 173 of the 233 journals and conference proceedings. The mean IPP is 4.57 citations per publication in the data set. The mean SJR is 2.11, with a standard deviation of 1.51, for the publication in the data set; and 650 of the publications have an SJR over 1. An expected citation score may be calculated by summing the IPP values for each publication, which equals 3,922 expected citations for the ADNI publications. Expected citations have steadily increased each year of the project since 2007. However, this set of ADNI publications collected an observed 17,854 citations, indicating a difference of 13,932 citations, or 3.55 times the expected citation count. Of ADNI publications, 481 (56%) had citation counts greater than the journal’s expected citation count; with the significant portion of the citations coming between 2008 and 2012.

**Fig 2:**
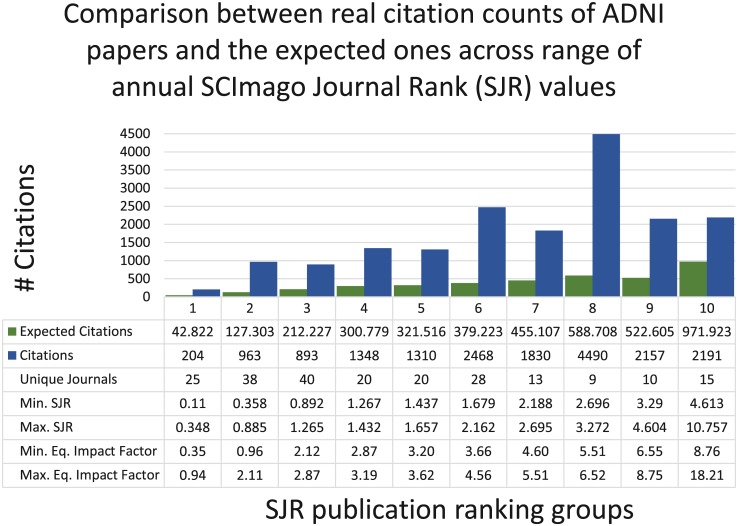
Plot of citation counts. The combined bar and line graph compares the expected citation counts (green bars) and total article citation counts (blue bars) though May 2015 for publications grouped by their annual journal SRJ publication groups (see S1 Table for detailed information about each group). Expected citations were calculated by multiplying the corresponding annual journal IPP score by the number of publications, and summing the totals for each SJR group. Total citations are calculated as the sum of citation counts provided by the Scopus database at the date of retrieval. The number of unique journals per group and the minimum and maximum SJR ranks are provided, as are equivalent Impact Factor scores calculated using a predictive equation generated by regression analysis of ADNI venue Impact Factor and SJR values between 2003 and 2015.

Fig 2 provides further descriptive statistics of the citation analysis by grouping publications by their annual SJR Rankings. S1 Table provides examples of journals from each of the groups. S3 Fig demonstrates the equivalence of SJR values to Impact Factor journal rankings: (A) A comparison of the normalized distributions of journal rank values shows significant overlap; (B) a line fit plot of the regression analysis of annual SJR values and Impact Factors values shows that on average, journals SJR values are 2.06 less than Impact Factors; and specific high ranking neurology journals are shown to be over ranked by Impact Factors by two standard deviations.

Fig 2 also shows a plot of the expected citations versus actual citations to ADNI publications for groups of journals with similar SJR values. Here the equivalent Impact Factor values are shown in the associated table, and they are calculated using the model produced by the regression analysis described above. While these results point to the strong citation impact of ADNI publications, the citation counts do not take into account the source of each citation. To address this issue, we did further analysis to show which disciplines are benefiting from the ADNI project. S4 Fig shows the trend plots of SJR rankings for journals publishing at least 5 ADNI articles over at least 3 years. The journal rank and publication trends are split out by the main subject area of a journal, and are shown over the years. The strength of the affiliation and collaboration networks within ADNI could result in strong intra-group citation patterns that account for the large difference in expected and received citations.

### Where

A geospatially plotted co-occurrence network was created to show the geographic distribution of collaborations between institutions with publications that use the ADNI data. The analysis identified 814 affiliated institutions nodes in the publication data, with 709 nodes are in the largest connect subnetwork (also called giant component), with 10,247 edges. ADNI affiliates have a high level of collaborations across the publications. Affiliate nodes have an average degree of 25.243, an average clustering coefficient of .838, and a network density of .031. The average path length for affiliates is 2.76, with a network diameter of 7.

**Fig 3:**
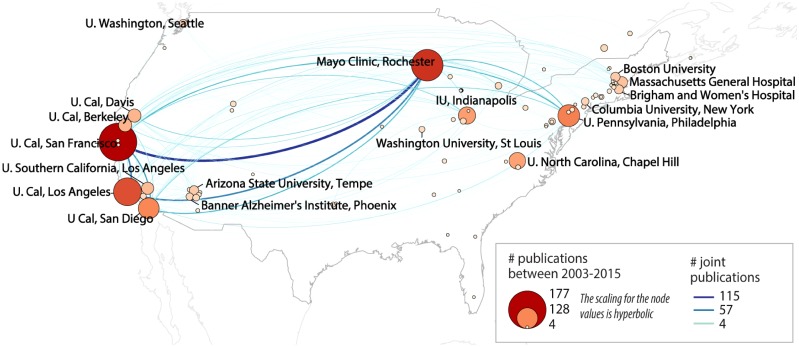
Geospatial map of publication co-occurrence network. Co-affiliation network overlaid on a geospatial map shows collaborating organizations affiliated with ADNI in North American based on co-authored publications. Only organizations with at least 4 publications are shown; organizations with at least 30 publications or that are a Core ADNI research institution have been labeled in the network. Organization relationships (edges) with four or more co-authorships are shown.

Fig 3 shows a portion of the full institution collaboration network, including only collaborations within North America that have at least 5 publications in the data set. The network shows 152 nodes and 448 edges. The average degree for this network is 5.895, and the average clustering coefficient is 0.784. The network diameter is 5, with an average path length of 2.598, and density of 0.034. S7 Fig shows an expanded geospatial map including North America and Europe.

To check whether the collaboration is related to the geospatial distance, we calculated the correlation between the geospatial distance of two institutions and their network connection (i.e., indicating the number of collaborative papers between them). The resulting Pearson correlation is -0.048, suggesting that the collaboration does not tend to be geospatial distance related.

### What over time

**Fig 4:**
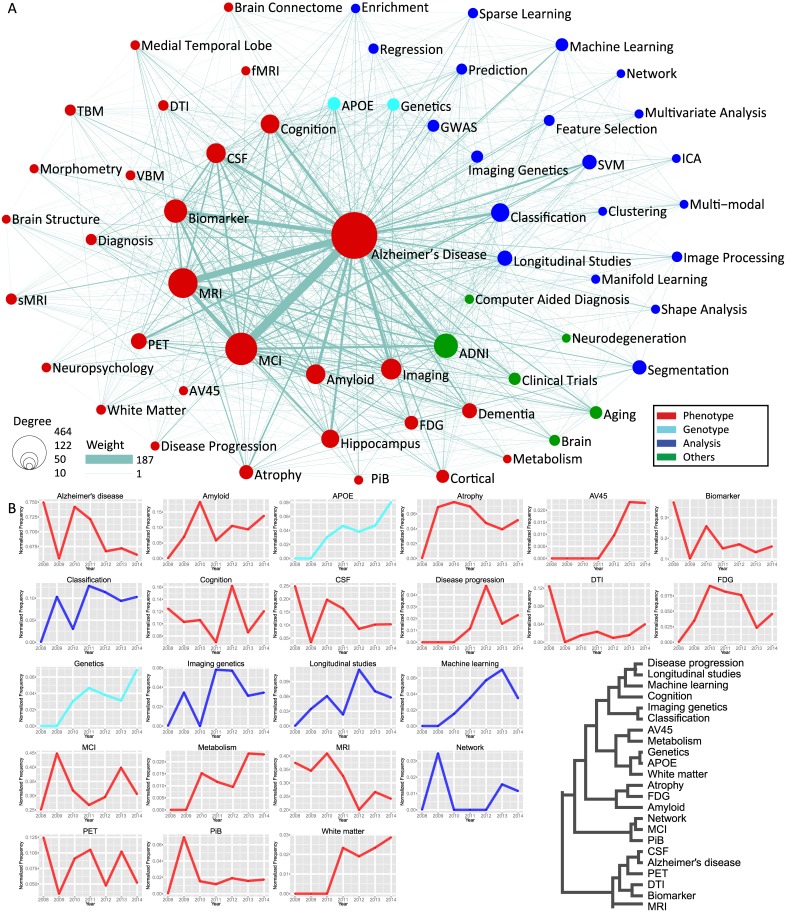
Keyword co-occurrence networks. (A) Keyword co-occurrence network containing only nodes with degree ≥ 10. Nodes represent keywords, and edges denote the joint appearance of keywords in a publication. Only nodes with degree ≥ 10 are shown. Both nodes and edges are scaled proportionally based on Bezier curve. Nodes are colored based on their categories: genotype, phenotype, analysis, and others. (B) Temporal profiles of selected keywords show the normalized frequency (frequency of keywords divided by number of publications) from 2008 to 2014. Hierarchical plot indicates clustering of selected keywords.

Two topical visualizations, keyword co-occurrence networks and topical temporal evolution, were constructed to show the co-occurrence and temporal development of selected ADNI topics. In total, there are 6,626 links identified among 939 keywords. Fig 4(A) shows the filtered keyword co-occurrence network in which only keywords with degree ≥ 10 are included. In Fig 4(A), links and nodes are sized proportional to their weights and degrees respectively. Keywords with degree ≥ 10 are labeled and categorized into four specific areas with difference colors, including phenotype, genotype, analysis and others. ADNI consortium consists of multiple cores focusing on various aspects of data and analysis, including biomarker, biostatistics, clinical, genetics, informatics, MRI, neuropathology, and PET. Most keywords from publications are associated with one or more of these cores. The dense connections among both intra- and inter-category keywords demonstrate the independent and more importantly collaborative efforts and impacts of these ADNI core areas.

In Fig 4(A), a few hub keywords maintain connections not only among themselves, but also with most remaining keywords. These turn out to be the primary and hot research topics, including “Alzheimer’s Disease”, “MCI”, “MRI”, “Biomarker”, “Imaging”, “Amyloid”, “CSF”, “Cognition”, “*APOE*”, “Genetics”, “Classification”, “Longitudinal studies”, and so on. Most of the other keywords are peripheral ones, with much smaller number of connections to other nodes. S5 Fig shows a subnetwork containing keywords belonging to major ADNI themes, including MRI, PET, other biological biomarkers, clinical and neuropsychological assessment, genetics, and disease and progression.

Research topics change over time. These changes, either active or inactive, can help guide researchers to identify driving demands of AD and other dementia studies across a spectrum of diverse, though interconnected topics. Fig 4(B) shows temporal profiles of 23 domain expert selected AD topics covering the categories of phenotype, genotype, and analysis, to indicate major AD research themes. Normalized frequencies of selected keywords from 2008 to 2014 illustrate the trajectories of AD research topics in the temporal dimension, and identify topics that have been continuously popular over the past decade in ADNI publications. “Alzheimer’s Disease”, as the major research focus of ADNI, gains the highest normalized frequency among all keywords. “MCI”-Mild Cognitive Impairment-an intermediate stage between the expected cognitive decline of normal aging and the more pronounced decline of dementia, is identified as the second hottest topic. A number of biomarkers have been widely investigated in ADNI studies including magnetic resonance imaging (MRI), positron emission tomography (PET), other biological markers, and clinical and neuropsychological assessment, to detect AD at a pre-dementia stage. From the temporal profile, the frequency of term “Biomarker” shows a large fluctuation between 2008 and 2010 before stabilizing after 2011. Several specific topics clustered together with “Biomarker”, including “MRI”, “CSF”, “PET” and “DTI”, show their similar trends of being examined.

There are various trends can be observed from the temporal profiles of topics. A few topics show a growing trend over time and burst at certain years. For example, “Amyloid” and “CSF” burst in 2010; frequencies of “PiB” and “Network” increase sharply in 2009; and “Disease progression” and “Longitudinal studies” show similar patterns and burst in 2012. Some other topics display approximately ever-increasing trends across years except very few transitory falls, including “*APOE*”, “AV45”, “Genetics”, “White matter”, “Metabolism”, “Imaging genetics” and “Machine learning”.

These trends demonstrate the impact of ADNI project in relevant research fields. For instance, “Genetics” and “*APOE*” show very similar and increasing trends from 2009 to 2014, indicating the growing impact of the genetics core established in 2008. The co-clustering pattern of “Longitudinal studies” and “Disease progression” shown in the dendrogram illustrates the study trend on the topic of AD progression. In addition, with the advance of multimodal imaging and high throughput genotyping techniques, “Imaging genetics”-a multidisciplinary research field that integrates imaging and genetics-displays an increasing trend, accompanied by relevant topics including various imaging modalities, genetics as well as analytical algorithms. Besides, S5 Fig shows a sub-network of keyword co-occurrences focusing on major ADNI themes including MRI, PET, other biological markers, genetics, clinical and neuropsychological assessment, disease and progression, and terms that across two or more domains.

### With whom over time

**Fig 5:**
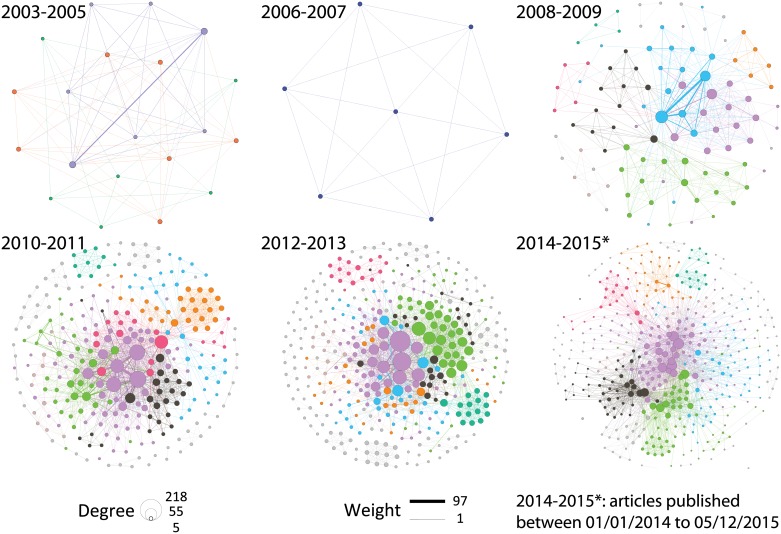
Growth of co-publication networks over time. Nodes represent institutions. Nodes are colored based on modularity and sized proportional to the natural logarithm of their degrees. Edges represent co-occurrence of institutions in publications, and are sized proportional to the number of co-publications. See S8 Fig for detailed sub networks containing only hub institutions with degree ≥ 30.

Co-publication networks were analyzed based on six time periods: the first period included 2003-2005 due to the limited number of early ADNI publications, and all the remaining ones are the biennial periods between 2006 and 2015. Fig 5 shows all the six co-publication networks, where nodes represent research institutions, and edges linking pairs of nodes represent their co-occurrences in the publications. Modularity analysis was performed on each network. Nodes are colored by the modules they belong to, and sized proportional to the natural logarithm of their degrees. Edges are weighted according to the number of co-publications. We also extracted sub-networks containing only hub institutions with degree ≥ 30, and showed those in S8 Fig. Shown in S6 Fig are the detailed statistics of co-publication networks over time. In S6 Fig, blue bars represent the number of unique edges: if there are multiple co-publications (i.e., edge weight > 1) between two institutions, we only count this collaboration/edge once. Orange bars correspond to the sum of edge weights: we accumulate the actual number of co-publications between each pair of institutions. The increasing trend of collaboration is very apparent (Fig 5 and S6 Fig, which is positively correlated with the growth of publications and institutions involved in the ADNI publications (Fig 1(A) and 1(B)).

From Fig 5, the number of nodes with high degrees grows rapidly over years, indicating that more and more institutions are expanding their ADNI collaborations with many other institutions. Co-publication network of 2003-2005 is denser than 2006-2007, although there are only 4 papers published in either 2003-2005 or 2006-2007. That is because in 2006-2007, one paper has no author information, and one paper has only one author and one affiliation; while all 4 papers in 2003-2005 contain multiple authors and multiple affiliations. Moreover, from 2010 through 2015, the number of low degree nodes greatly increased, which demonstrates more research institutions are analyzing the ADNI data. However, the average degree of institutions (blue and orange bars in S6(B) Fig) does not show significant increase since 2010. One possible reason is that the increased collaborations occur more among newly involved nodes, or between newly involved and existing high-degree nodes, but less among high-degree nodes. This reason is demonstrated by the statistics on edges (S6(A) Fig), where the numbers of unique edges (blue bars) and weighted edges (orange bars) both increased over time while their differences do not. These statistics indicate that the increases in edges are more often distributed in newly occurring co-publications. In addition, compared to 0.5% average connectivity percentage of typical collaboration networks, ADNI studies from 2008 to 2015 yield an average connectivity percentage of 4.5% (with minimum of 2.3% and maximum of 9.2%). This indicates that the ADNI studies are more collaborative than typical studies on average. All the above implicates that the ADNI resource not only facilitates collaborative studies involving more and more research institutions, but also enables many newly collaborations that may cross multiple disciplines. These new collaborations have the potential to yield innovative thoughts and findings.

## Discussion and outlook

ADNI research literature has greatly expanded over the past decades. Existing surveys or reviews mostly focus on summarizing the scientific findings in the usage of ADNI data. It is still an under-explored territory to assess the scientific impact and growth of the ADNI results systematically. To promote the information communication of ADNI studies, this paper provides a comprehensive evidence of scientific growth and impact of the ADNI results over a period of 12 years, as measured by relevant statistics and as visualized from different aspects.

The following are a few significant observations: 1) the number of ADNI publications increased linearly from 2008, so did the number of the involved institutions. 2) ADNI publications yielded significantly stronger citation impact than publications from the same set of journals. 3) Collaborations between institutions with publications using the ADNI data were prominently high, and significantly increased over the time. 4) Research topics of the ADNI related studies as presented by keywords evolved over the times, while dense interactions existed among them indicating the interdisciplinary nature of the studied topics partially represented by the themes of the ADNI cores.

Both statistical results and visualizations demonstrate the increasing attention of AD research, strong citation impact of ADNI publications, the expanding collaboration networks among researchers, institutions and ADNI core areas, and the evolution of ADNI research topics. These findings reflect the significance and achievement of the ADNI project, shows the increasing attention of ADNI-related research, indicate the trend of the AD research and potential hot topics, and greatly improve the information communication. All findings from this paper show the collaborative culture and high productivity of the ADNI community, as well as the profound impact of the availability of the ADNI database on promoting AD research forward rapidly.

When comparing the growth of co-publications over time, more newly-established collaborations occur while not very significantly reflected in already existed ones. This can inspire innovative thoughts through developing new cooperation. However, closer cooperation is also with the same importance as good foundation can improve the conduct and efficacy.

ADNI project has been running for more than 12 years, from the initial study termed ADNI-1, to ADNI-GO, ADNI-2, and then newly started ADNI-3. Each new phase typically studies a subset of continuing participants plus a set of new participants, examines more imaging and other biomarkers, and collects the longitudinal profiles of the participants. The significant and growing impact of ADNI publications can be contributed in part by the enrolling of more data and samples, the employment of innovative technologies, and the increasing of public awareness of AD and other dementias. Given that there is no big difference on the participating ADNI centers and study sites across all four ADNI phases, the observed increase on the number of involved institutions and inter-institutional collaborations is mostly attributed to the growing base of researchers analyzing the ADNI data and thanks to the open science nature of the ADNI project.

Recent ADNI-related studies continue and expand to the investigation of novel phenotypes, epigenetics, blood RNA and so on, and have presented promising discoveries. We have reviewed all the ADNI genetics-related publications as of 12/31/2016 [39]. The results have demonstrated the achievement of ADNI Genetics core, which is designed to provide genetic resources and facilitate research opportunities in qualitative and quantitative genetics. A future direction we plan to pursue is to apply and expand similar analysis to cover all the ADNI themes, including imaging, biomarker, cognition and other phenotypes, as well as analysis approaches in addition to genetics, for illustrating more comprehensive scientific progress and impact of the ADNI project.

## Supporting information

S1 Fig Number of publications versus author position. (A) Number of publications versus author position: For each point, the x-axis value indicates the position of an author, and the y-axis value is the number of papers this author published at this author rank. For example, an author with three first author papers and one second author paper contributes two points (1,3) and (2,1) to the plot. (B) Number of publications versus reversed author position: Analogous to (A) except that author position is ranked reversely. For example, an author with one last author paper and four second last author papers contributes two points (1,1) and (2,4) to the plot.
(DOCX)

S2 Fig Top journals of ADNI publications. Top journals or conference proceedings ranked by the number of ADNI publications. Wordle visualization in top-right lists top and other journals size and color coded by the number of ADNI publications.
(DOCX)

S3 Fig Correlation of SJR to Impact Factor for ADNI publication journals. (A) compares the distribution of the normalized SCImago Journal Rank (SJR) and Impact Factors (J_rank_ − mean(J_rank_)/StDev(J_rank_)). (B) is a line fit plot of regression analysis of journal SJR and Impact Factor values that are within two standard deviations of the mean difference between SJR and Impact Factor values; the trend line for the plot is a power function with an R-squared of 0.819.
(DOCX)

S4 Fig Annual SCImago Journal Rankings and annual citation counts. Plot of shows annual SCImago Journal Rankings and annual citation counts for the journals that have published at least 5 papers related to ADNI in at least 3 years, between 2006 and 2014. Journals are grouped by a heuristic categorization system derived from the Scopus journal subject categories. The size of the ring symbol indicates the number of publications per year and the color of the ring indicates the number of citations per year on a log scale. The journals are: a—*Journal of Neuroscience*; b—*Brain*; c—*Annals of Neurology*; d—*JAMA Neurology // Archives of Neurology*; e—*Neurology*; f—*Acta Neuropathologica*; g—*Dementia and Geriatric Cognitive Disorders*; h—*Journal of Alzheimer’s Disease*; i—*Alzheimer’s Research and Therapy*; j—*Current Alzheimer Research*; k—*Alzheimer Disease and Associated Disorders*; l—*Alzheimer’s and Dementia*; m—*NeuroImage*; n—*Human Brain Mapping*; o—*American Journal of Neuroradiology*; p—*Brain Imaging and Behavior*; q—*NeuroImage: Clinical*; r—*Journal of Nuclear Medicine*; s—*IEEE Transactions on Medical Imaging*; t -*Medical Image Analysis*; u—*Neurobiology of Aging*; v—*Frontiers in Aging Neuroscience*; w—*American Journal of Geriatric Psychiatry*; x—*International Journal of Geriatric Psychiatry*; y—*Molecular Psychiatry*; z—*Lecture Notes in Computer Science*; aa—*PLoS ONE*.
(DOCX)

S5 Fig Keyword co-occurrence network focused on major ADNI themes. Nodes represent keywords relevant to major ADNI themes, including MRI, PET, other biological biomarkers, clinical and neuropsychological assessment, genetics, and disease and progression. Edges denote the joint appearance of keywords in a publication. Nodes are colored based on the themes they belonged to, and those across three or more themes are colored in dark blue. Both nodes and edges were scaled proportionally based on Bezier curve. Only nodes with degree > 2 are shown.
(DOCX)

S6 Fig Statistics of co-publication networks. (A) Number of co-publications over time, that is, the total number of edges (blue bar) or the sum of edge weights (orange bar) over years. Blue bars represent the number of unique edges: if there are multiple co-publications (i.e., edge weight > 1) between two institutions, the collaboration/edge is counted only once. Orange bars represent the sum of all collaborations (non-unique) between institutions. (B) Degree of co-publications over time, which is the average node degree in each co-publication network. Blue bars are calculated based on the degrees using edge counts, and orange bars are based on the weighted degrees using edge weights.
(DOCX)

S7 Fig World-wide geospatial map of publication co-occurrence network. Co-affiliation network overlaid on a geospatial map shows collaborating organizations affiliated with ADNI in world-wide based on co-authored publications. Only organizations with at least 4 publications are shown; organizations with at least 30 publications or that are a Core ADNI research institution have been labeled in the network. Organization relationships (edges) with four or more co-authorships are shown.
(DOCX)

S8 Fig Sub-network of hubs from co-publication networks over time. Sub-networks were extracted from the full co-publication networks, where only nodes with degree ≥ 30 were included. Both size and color of node were from the original co-publication network. Node were labeled by the institution name.
(DOCX)

S1 Table SJR Groups. For each SJR Groups, five example journals from each group were selected based on the total number of publications that they received.
(DOCX)

S1 Dataset Processed and cleaned ADNI publication citation data, including those used for visualization. (XLSX)
