# Supplementary material for: Mapping longitudinal scientific progress, collaboration and impact of the Alzheimer’s disease neuroimaging initiative
Source: PLoS One. 2017 Nov 2;12(11):e0186095. doi: 10.1371/journal.pone.0186095 (PMC5667864; doi:10.1371/journal.pone.0186095)
Supplement: S1 Fig — (A) Number of publications versus author position: For each point, the x-axis value indicates the position of an author, and the y-axis value is the number of papers this author published at this author rank. For example, an author with three first author papers and one second author paper contributes two points (1,3) and (2,1) to the plot. (B) Number of publications versus reversed author position: Analogous to (A) except that author position is ranked reversely. For example, an author with one last author paper and four second last author papers contributes two points (1,1) and (2,4) to the plot. (DOCX) [file pone.0186095.s001.docx]

**Supplementary Materials for "** **Mapping longitudinal scientific progress, collaboration and impact of the Alzheimer’s disease neuroimaging initiative " by Xiaohui Yao, Jingwen Yan, Michael Ginda, Katy Börner, Andrew J. Saykin, Li Shen, for the Alzheimer's disease neuroimaging initiative.**

**S1 Fig. Number of publications versus author position.** (A) Number of publications versus author position: For each point, the x-axis value indicates the position of an author, and the y-axis value is the number of papers this author published at this author rank. For example, an author with three first author papers and one second author paper contributes two points (1,3) and (2,1) to the plot. (B) Number of publications versus reversed author position: Analogous to (A) except that author position is ranked reversely. For example, an author with one last author paper and four second last author papers contributes two points (1,1) and (2,4) to the plot.
