# Supplementary material for: Mapping longitudinal scientific progress, collaboration and impact of the Alzheimer’s disease neuroimaging initiative
Source: PLoS One. 2017 Nov 2;12(11):e0186095. doi: 10.1371/journal.pone.0186095 (PMC5667864; doi:10.1371/journal.pone.0186095)
Supplement: S2 Fig — Top journals or conference proceedings ranked by the number of ADNI publications. Wordle visualization in top-right lists top and other journals size and color coded by the number of ADNI publications. (DOCX) [file pone.0186095.s002.docx]

**Supplementary Materials for "** **Mapping longitudinal scientific progress, collaboration and impact of the Alzheimer’s disease neuroimaging initiative " by Xiaohui Yao, Jingwen Yan, Michael Ginda, Katy Börner, Andrew J. Saykin, Li Shen, for the Alzheimer's disease neuroimaging initiative.**


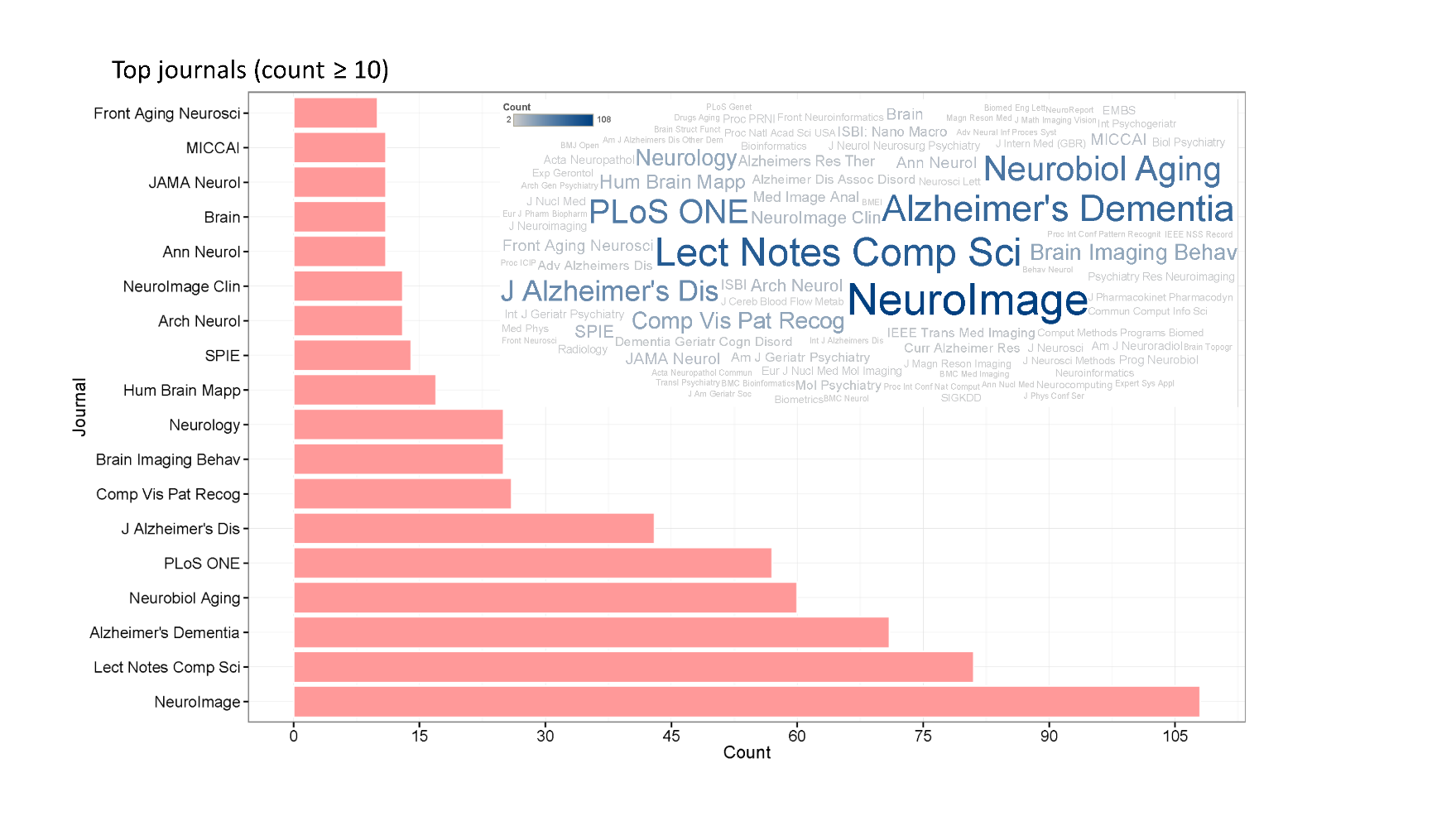


**S2 Fig.** **Top journals of ADNI publications.** Top journals or conference proceedings ranked by the number of ADNI publications. Wordle visualization in top-right lists top and other journals size and color coded by the number of ADNI publications.
