# Supplementary material for: Mapping longitudinal scientific progress, collaboration and impact of the Alzheimer’s disease neuroimaging initiative
Source: PLoS One. 2017 Nov 2;12(11):e0186095. doi: 10.1371/journal.pone.0186095 (PMC5667864; doi:10.1371/journal.pone.0186095)
Supplement: S3 Fig — (A) compares the distribution of the normalized SCImago Journal Rank (SJR) and Impact Factors (Jrank − mean(Jrank)/StDev(Jrank)). (B) is a line fit plot of regression analysis of journal SJR and Impact Factor values that are within two standard deviations of the mean difference between SJR and Impact Factor values; the trend line for the plot is a power function with an R-squared of 0.819. (DOCX) [file pone.0186095.s003.docx]

**Supplementary Materials for "** **Mapping longitudinal scientific progress, collaboration and impact of the Alzheimer’s disease neuroimaging initiative " by Xiaohui Yao, Jingwen Yan, Michael Ginda, Katy Börner, Andrew J. Saykin, Li Shen, for the Alzheimer's disease neuroimaging initiative.**

**S3 Fig. Correlation of SJR to Impact Factor for ADNI publication journals.** (A) compares the distribution of the normalized SCImago Journal Rank (SJR) and Impact Factors (J_rank_-J_meanrank_/StDev(J_rank_). (B) is a line fit plot of regression analysis of journal SJR and Impact Factor values that are within two standard deviations of the mean difference between SJR and Impact Factor values; the trend line for the plot is a power function with an R-squared of 0.819.
