# Supplementary material for: Mapping longitudinal scientific progress, collaboration and impact of the Alzheimer’s disease neuroimaging initiative
Source: PLoS One. 2017 Nov 2;12(11):e0186095. doi: 10.1371/journal.pone.0186095 (PMC5667864; doi:10.1371/journal.pone.0186095)
Supplement: S4 Fig — Plot of shows annual SCImago Journal Rankings and annual citation counts for the journals that have published at least 5 papers related to ADNI in at least 3 years, between 2006 and 2014. Journals are grouped by a heuristic categorization system derived from the Scopus journal subject categories. The size of the ring symbol indicates the number of publications per year and the color of the ring indicates the number of citations per year on a log scale. The journals are: a—Journal of Neuroscience; b—Brain; c—Annals of Neurology; d—JAMA Neurology // Archives of Neurology; e—Neurology; f—Acta Neuropathologica; g—Dementia and Geriatric Cognitive Disorders; h—Journal of Alzheimer’s Disease; i—Alzheimer’s Research and Therapy; j—Current Alzheimer Research; k—Alzheimer Disease and Associated Disorders; l—Alzheimer’s and Dementia; m—NeuroImage; n—Human Brain Mapping; o—American Journal of Neuroradiology; p—Brain Imaging and Behavior; q—NeuroImage: Clinical; r—Journal of Nuclear Medicine; s—IEEE Transactions on Medical Imaging; t -Medical Image Analysis; u—Neurobiology of Aging; v—Frontiers in Aging Neuroscience; w—American Journal of Geriatric Psychiatry; x—International Journal of Geriatric Psychiatry; y—Molecular Psychiatry; z—Lecture Notes in Computer Science; aa—PLoS ONE. (DOCX) [file pone.0186095.s004.docx]

**Supplementary Materials for "** **Mapping longitudinal scientific progress, collaboration and impact of the Alzheimer’s disease neuroimaging initiative " by Xiaohui Yao, Jingwen Yan, Michael Ginda, Katy Börner, Andrew J. Saykin, Li Shen, for the Alzheimer's disease neuroimaging initiative.**


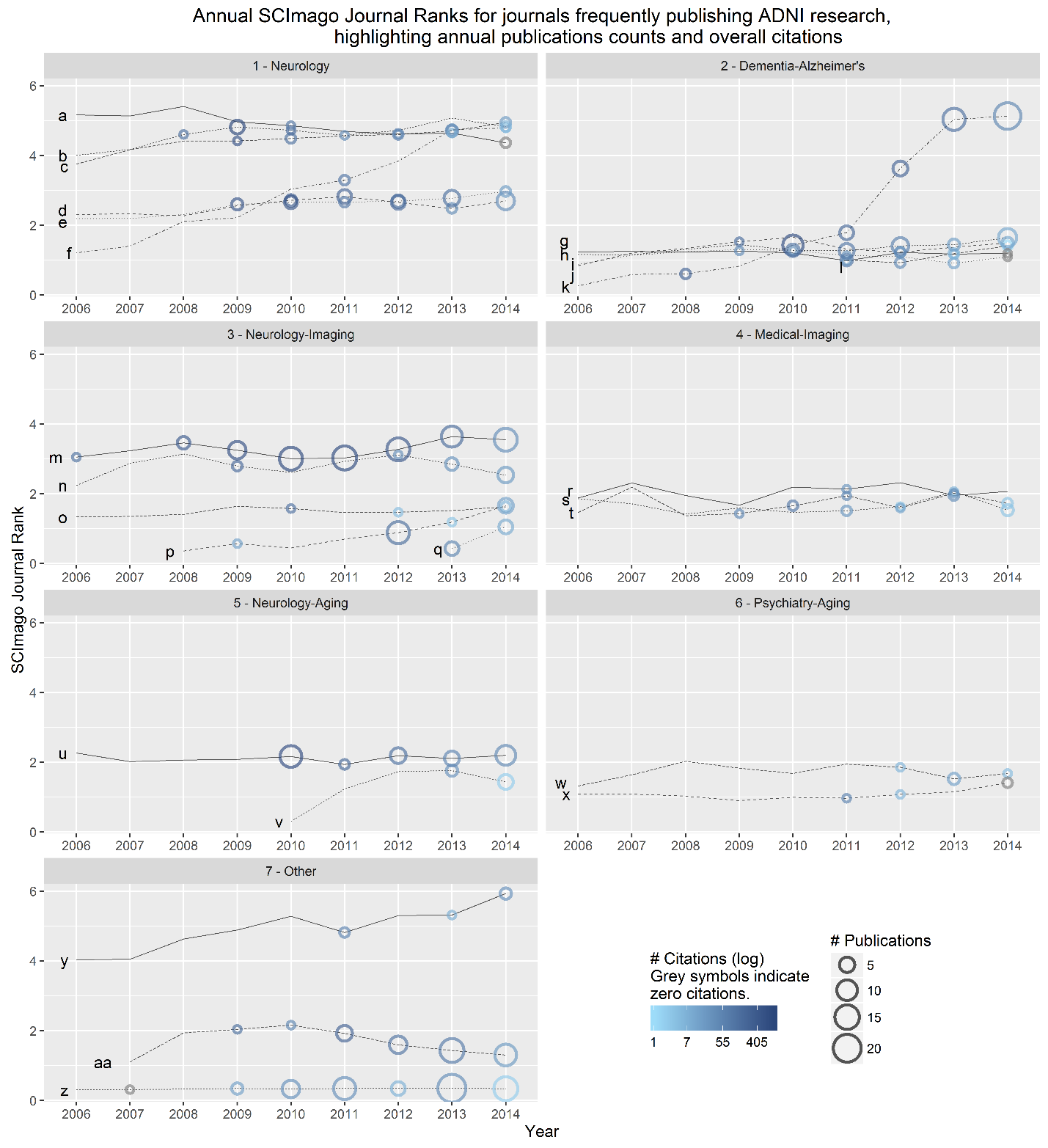


**S4 Fig. Annual SCImago Journal Rankings and annual citation counts.** Plot of shows annual SCImago Journal Rankings and annual citation counts for the journals that have published at least 5 papers related to ADNI in at least 3 years, between 2006 and 2014. Journals are grouped by a heuristic categorization system derived from the Scopus journal subject categories. The size of the ring symbol indicates the number of publications per year and the color of the ring indicates the number of citations per year on a log scale. The journals are: a - Journal of Neuroscience; b - Brain; c - Annals of Neurology;
d - JAMA Neurology // Archives of Neurology; e - Neurology; f - Acta Neuropathologica;
g - Dementia and Geriatric Cognitive Disorders; h - Journal of Alzheimer's Disease;
i - Alzheimer's Research and Therapy; j - Current Alzheimer Research; k - Alzheimer Disease and Associated Disorders; l - Alzheimer's and Dementia; m - NeuroImage;
n - Human Brain Mapping; o - American Journal of Neuroradiology;
p - Brain Imaging and Behavior; q - NeuroImage: Clinical; r - Journal of Nuclear Medicine;
s - IEEE Transactions on Medical Imaging; t - Medical Image Analysis;
u - Neurobiology of Aging; v - Frontiers in Aging Neuroscience;
w - American Journal of Geriatric Psychiatry; x - International Journal of Geriatric Psychiatry;
y - Molecular Psychiatry; z - Lecture Notes in Computer Science; aa - PLoS ONE.
