# Supplementary material for: Mapping longitudinal scientific progress, collaboration and impact of the Alzheimer’s disease neuroimaging initiative
Source: PLoS One. 2017 Nov 2;12(11):e0186095. doi: 10.1371/journal.pone.0186095 (PMC5667864; doi:10.1371/journal.pone.0186095)
Supplement: S5 Fig — Nodes represent keywords relevant to major ADNI themes, including MRI, PET, other biological biomarkers, clinical and neuropsychological assessment, genetics, and disease and progression. Edges denote the joint appearance of keywords in a publication. Nodes are colored based on the themes they belonged to, and those across three or more themes are colored in dark blue. Both nodes and edges were scaled proportionally based on Bezier curve. Only nodes with degree > 2 are shown. (DOCX) [file pone.0186095.s005.docx]

**Supplementary Materials for "Mapping longitudinal scientific progress, collaboration and impact of the Alzheimer’s Disease Neuroimaging Initiative (ADNI)" by Xiaohui Yao, Jingwen Yan, Michael Ginda, Katy Börner, Andrew J. Saykin, Li Shen, for the Alzheimer's Disease Neuroimaging Initiative.**


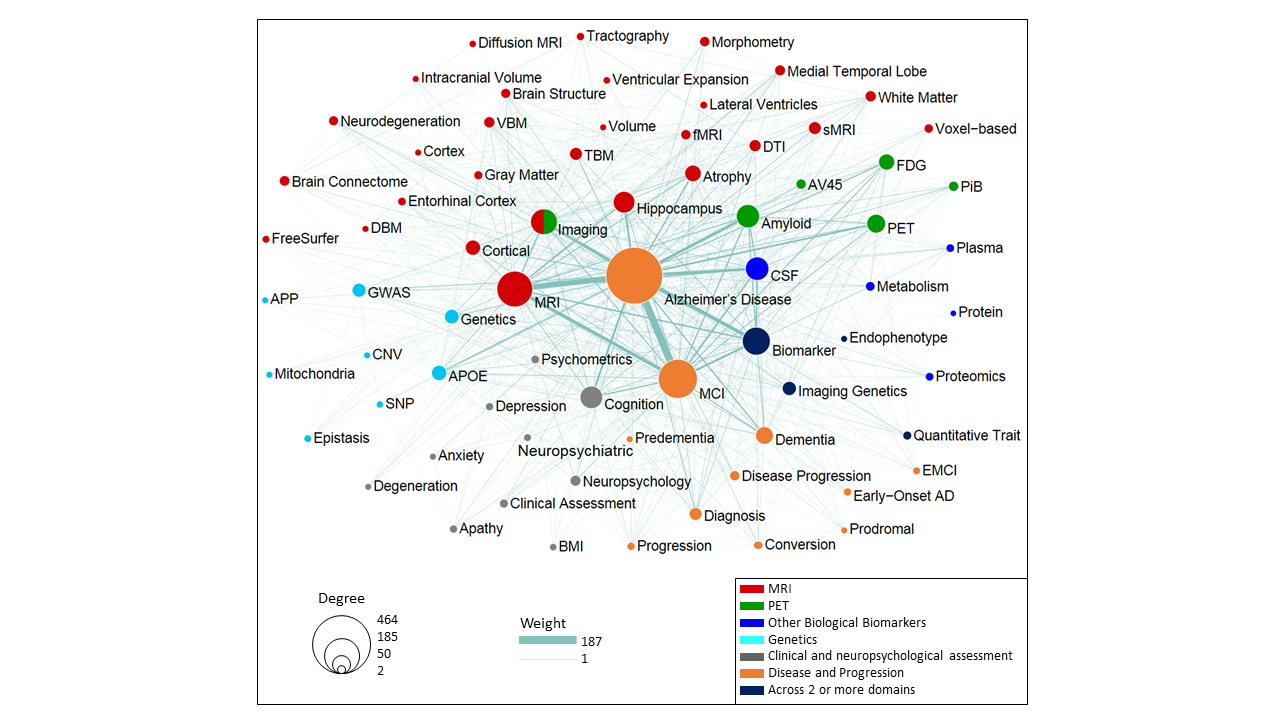


**S5 Fig.** **Keyword co-occurrence network focused on major ADNI themes.** Nodes represent keywords relevant to major ADNI themes, including MRI, PET, other biological biomarkers, clinical and neuropsychological assessment, genetics, and disease and progression. Edges denote the joint appearance of keywords in a publication. Nodes are colored based on the themes they belonged to, and those across three or more themes are colored in dark blue. Both nodes and edges were scaled proportionally based on Bezier curve. Only nodes with degree > 2 are shown.
