# Supplementary material for: Mapping longitudinal scientific progress, collaboration and impact of the Alzheimer’s disease neuroimaging initiative
Source: PLoS One. 2017 Nov 2;12(11):e0186095. doi: 10.1371/journal.pone.0186095 (PMC5667864; doi:10.1371/journal.pone.0186095)
Supplement: S6 Fig — (A) Number of co-publications over time, that is, the total number of edges (blue bar) or the sum of edge weights (orange bar) over years. Blue bars represent the number of unique edges: if there are multiple co-publications (i.e., edge weight > 1) between two institutions, the collaboration/edge is counted only once. Orange bars represent the sum of all collaborations (non-unique) between institutions. (B) Degree of co-publications over time, which is the average node degree in each co-publication network. Blue bars are calculated based on the degrees using edge counts, and orange bars are based on the weighted degrees using edge weights. (DOCX) [file pone.0186095.s006.docx]

**Supplementary Materials for "Mapping longitudinal scientific progress, collaboration and impact of the Alzheimer’s Disease Neuroimaging Initiative (ADNI)" by Xiaohui Yao, Jingwen Yan, Michael Ginda, Katy Börner, Andrew J. Saykin, Li Shen, for the Alzheimer's Disease Neuroimaging Initiative.**

**
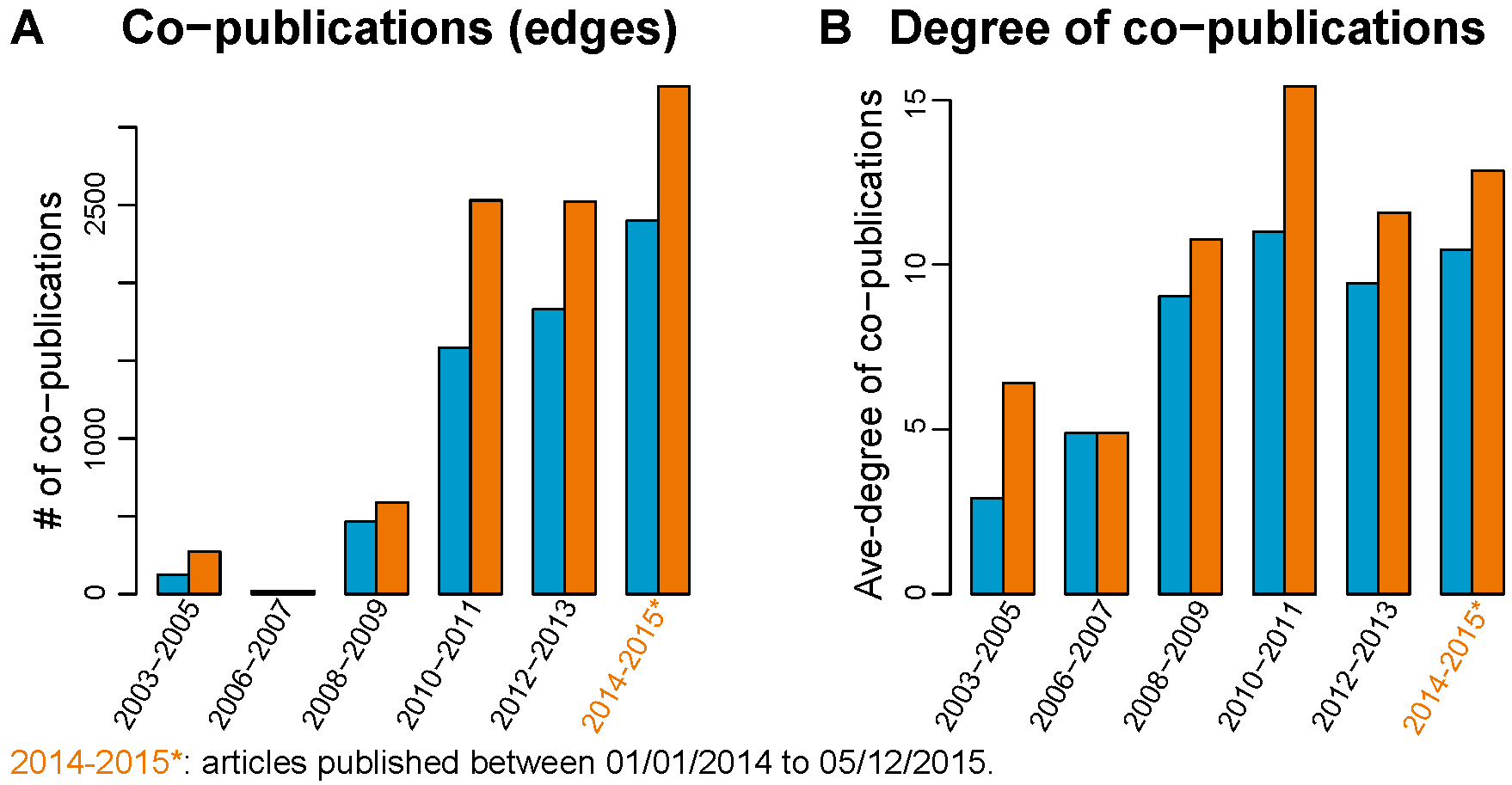
S6 Fig.** **Statistics of co-publication networks.** (A) Number of co-publications over time, that is, the total number of edges (blue bar) or the sum of edge weights (orange bar) over years. Blue bars represent the number of unique edges: if there are multiple co-publications (i.e., edge weight > 1) between two institutions, the collaboration/edge is counted only once. Orange bars represent the sum of all collaborations (non-unique) between institutions. (B) Degree of co-publications over time, which is the average node degree in each co-publication network. Blue bars are calculated based on the degrees using edge counts, and orange bars are based on the weighted degrees using edge weights.
