# Supplementary material for: Mapping longitudinal scientific progress, collaboration and impact of the Alzheimer’s disease neuroimaging initiative
Source: PLoS One. 2017 Nov 2;12(11):e0186095. doi: 10.1371/journal.pone.0186095 (PMC5667864; doi:10.1371/journal.pone.0186095)
Supplement: S7 Fig — Co-affiliation network overlaid on a geospatial map shows collaborating organizations affiliated with ADNI in world-wide based on co-authored publications. Only organizations with at least 4 publications are shown; organizations with at least 30 publications or that are a Core ADNI research institution have been labeled in the network. Organization relationships (edges) with four or more co-authorships are shown. (DOCX) [file pone.0186095.s007.docx]

**Supplementary Materials for "Mapping longitudinal scientific progress, collaboration and impact of the Alzheimer’s Disease Neuroimaging Initiative (ADNI)" by Xiaohui Yao, Jingwen Yan, Michael Ginda, Katy Börner, Andrew J. Saykin, Li Shen, for the Alzheimer's Disease Neuroimaging Initiative.**


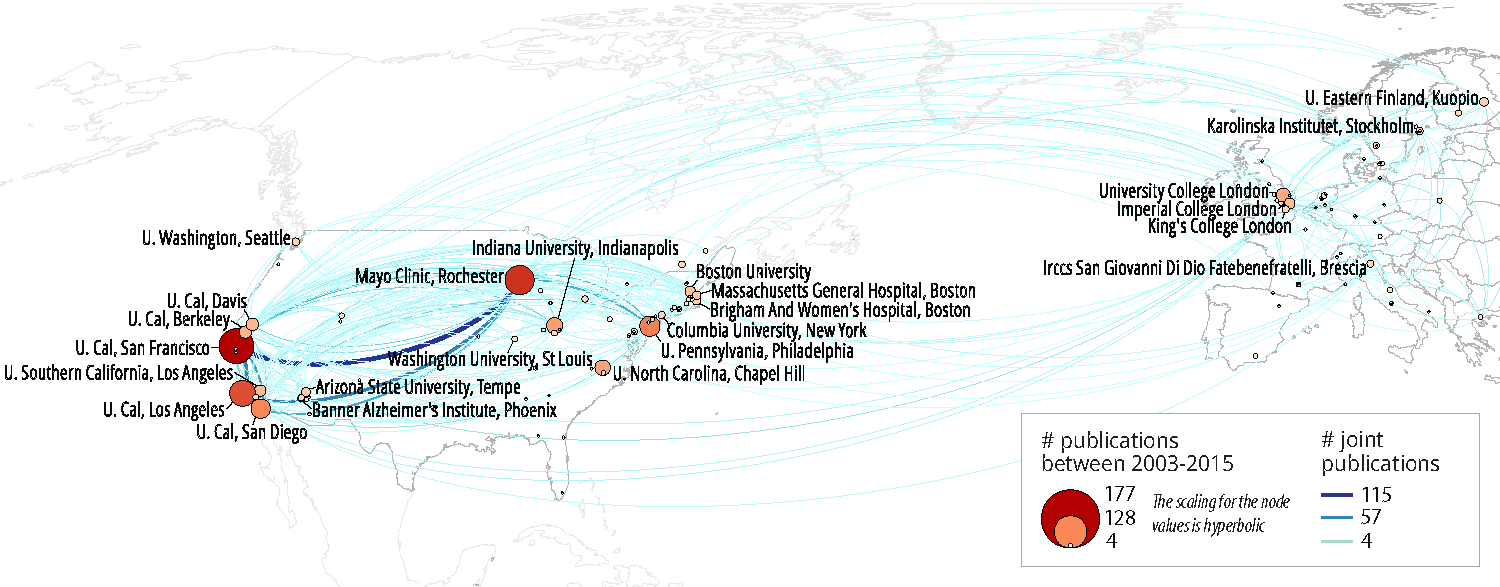


**S7 Fig.** **Geospatial map of publication co-occurrence network (including North America and Europe).** Co-affiliation network overlaid on a geospatial map shows collaborating organizations affiliated with ADNI in co-authored publications from North America and Europe. Only organizations with at least 4 publications are shown; organizations with at least 30 publications or that are core ADNI research institutions have been labeled in the network. Organization relationships (edges) with four or more co-authorships are shown.
