# Supplementary material for: Mapping longitudinal scientific progress, collaboration and impact of the Alzheimer’s disease neuroimaging initiative
Source: PLoS One. 2017 Nov 2;12(11):e0186095. doi: 10.1371/journal.pone.0186095 (PMC5667864; doi:10.1371/journal.pone.0186095)
Supplement: S8 Fig — Sub-networks were extracted from the full co-publication networks, where only nodes with degree ≥ 30 were included. Both size and color of node were from the original co-publication network. Node were labeled by the institution name. (DOCX) [file pone.0186095.s008.docx]

**Supplementary Materials for "Mapping longitudinal scientific progress, collaboration and impact of the Alzheimer’s Disease Neuroimaging Initiative (ADNI)" by Xiaohui Yao, Jingwen Yan, Michael Ginda, Katy Börner, Andrew J. Saykin, Li Shen, for the Alzheimer's Disease Neuroimaging Initiative.**


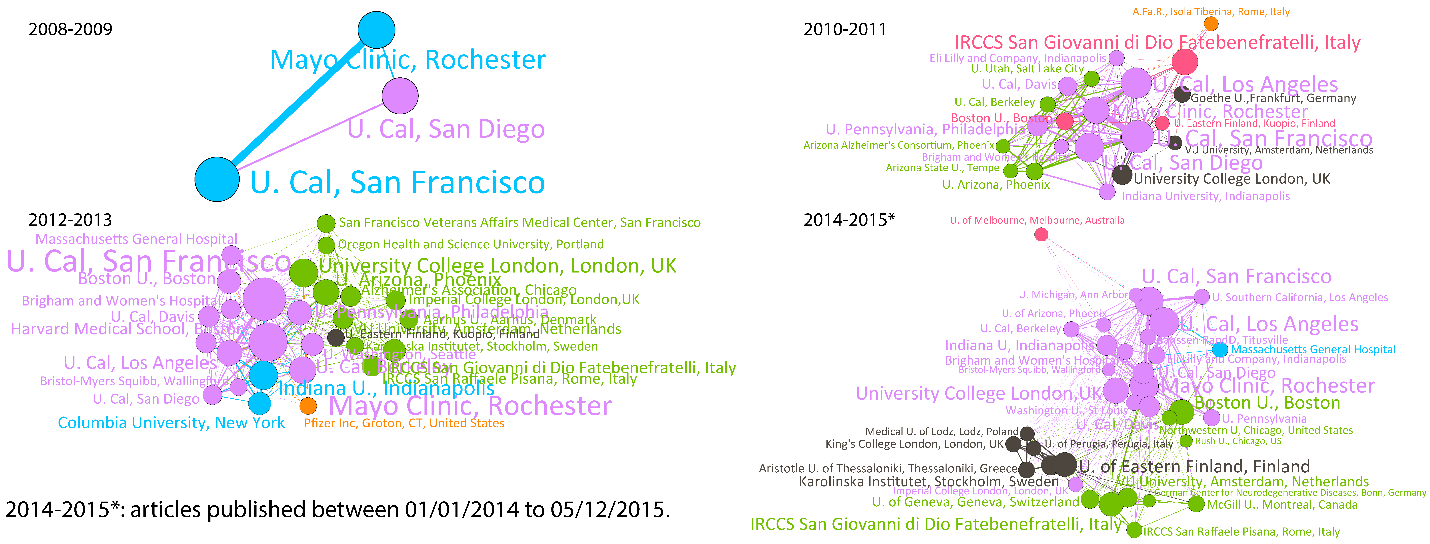


**S8 Fig. Sub-networks of hub institutions from co-publication networks over time.** Sub-networks were extracted from the full co-publication networks (see Fig. 5), where only nodes with degree ≥ 30 were included. Both the relative sizes and colors of nodes were the same as those from the original co-publication networks. Nodes were labeled by the institution names. No sub-networks were extracted from co-publication networks of 2003-2005 and 2006-2007, as there were no nodes with degree ≥ 30.
