# Supplementary material for: Mapping longitudinal scientific progress, collaboration and impact of the Alzheimer’s disease neuroimaging initiative
Source: PLoS One. 2017 Nov 2;12(11):e0186095. doi: 10.1371/journal.pone.0186095 (PMC5667864; doi:10.1371/journal.pone.0186095)
Supplement: S1 Table — For each SJR Groups, five example journals from each group were selected based on the total number of publications that they received. (DOCX) [file pone.0186095.s009.docx]

**Supplementary Materials for "** **Mapping longitudinal scientific progress, collaboration and impact of the Alzheimer’s disease neuroimaging initiative " by Xiaohui Yao, Jingwen Yan, Michael Ginda, Katy Börner, Andrew J. Saykin, Li Shen, for the Alzheimer's disease neuroimaging initiative.**

**S1 Table**. **SJR Groups.** For each SJR Groups, five example journals from each group were selected based on the total number of publications that they received.

| **SJR Group*** | **Min. SJR** | **Max. SJR** |
| --- | --- | --- |
| **Group 1** | **0.11** | **0.348** |
| Lecture Notes in Computer Science | 0.311 | 0.348 |
| Communications in Computer and Information Science | 0.145 | 0.148 |
| Proceedings - International Conference on Pattern Recognition | 0.196 | 0.196 |
| Biomedical Engineering Letters | 0.268 | 0.268 |
| IEEE Nuclear Science Symposium Conference Record | 0.211 | 0.259 |
| **Group 2** | **0.358** | **0.885** |
| Brain Imaging and Behavior | 0.571 | 0.885 |
| NeuroImage: Clinical | 0.43 | 0.43 |
| Journal of Neuroimaging | 0.656 | 0.701 |
| Journal of Pharmacokinetics and Pharmacodynamics | 0.492 | 0.84 |
| International Psychogeriatrics | 0.74 | 0.825 |
| **Group 3** | **0.892** | **1.265** |
| Alzheimer's Research and Therapy | 0.928 | 1.189 |
| NeuroImage: Clinical | 1.052 | 1.052 |
| Journal of Alzheimer's Disease | 1.265 | 1.265 |
| Alzheimer Disease and Associated Disorders | 0.914 | 1.132 |
| Dementia and Geriatric Cognitive Disorders | 0.983 | 1.224 |
| **Group 4** | **1.267** | **1.432** |
| PLoS ONE | 1.3 | 1.432 |
| Alzheimer's and Dementia | 1.428 | 1.428 |
| Journal of Alzheimer's Disease | 1.281 | 1.411 |
| International Journal of Geriatric Psychiatry | 1.409 | 1.409 |
| Psychiatry Research - Neuroimaging | 1.412 | 1.412 |
| **Group 5** | **1.437** | **1.657** |
| Journal of Alzheimer's Disease | 1.445 | 1.648 |
| PLoS ONE | 1.594 | 1.594 |
| Brain Imaging and Behavior | 1.657 | 1.657 |
| Frontiers in Aging Neuroscience | 1.437 | 1.437 |
| IEEE Transactions on Medical Imaging | 1.512 | 1.634 |
| **Group 6** | **1.679** | **2.162** |
| Neurobiology of Aging | 1.938 | 2.162 |
| PLoS ONE | 1.924 | 2.158 |
| Medical Image Analysis | 1.728 | 2.018 |
| Journal of Nuclear Medicine | 1.951 | 2.132 |
| Alzheimer's and Dementia | 1.788 | 1.788 |
| **Group 7** | **2.188** | **2.695** |
| Neurobiology of Aging | 2.188 | 2.199 |
| JAMA Neurology // Arch Neurol | 2.485 | 2.695 |
| Neurology | 2.601 | 2.693 |
| Human Brain Mapping | 2.535 | 2.535 |
| Journal of Cerebral Blood Flow and Metabolism | 2.415 | 2.415 |
| **Group 8** | **2.696** | **3.272** |
| NeuroImage | 3.006 | 3.272 |
| Neurology | 2.775 | 2.968 |
| JAMA Neurology // Arch Neurol | 2.715 | 2.826 |
| Human Brain Mapping | 2.793 | 3.117 |
| Radiology | 2.696 | 3.148 |
| **Group 9** | **3.29** | **4.604** |
| NeuroImage | 3.46 | 3.635 |
| Alzheimer's and Dementia | 3.628 | 3.628 |
| Progress in Neurobiology | 3.904 | 4.19 |
| Annals of Neurology | 4.417 | 4.604 |
| Bioinformatics | 3.576 | 4.259 |
| **Group 10** | **4.613** | **10.757** |
| Alzheimer's and Dementia | 5.039 | 5.129 |
| Brain | 4.722 | 4.826 |
| Molecular Psychiatry | 4.816 | 5.93 |
| Annals of Neurology | 4.711 | 4.946 |
| Acta Neuropathologica | 4.743 | 4.79 |
| * Journals may appear across groups due to annual changes in SJR ranking values. | | |
